# AI-driven analysis of diabetes risk determinants in U.S. adults: Exploring disease prevalence and health factors

**DOI:** 10.1371/journal.pone.0328655

**Published:** 2025-09-03

**Authors:** Dawid Majcherek, Antoni Ciesielski, Paweł Sobczak

**Affiliations:** 1 Department of International Management, Collegium of World Economy, SGH Warsaw School of Economics, Warsaw, Poland; 2 Technical Schools Complex named after Waldemar Gostomczyk in Ostrów Wielkopolski, Ostrów Wielkopolski, Poland; 3 Department of Technical Sciences, Faculty of Economics and Technical Sciences, University of Applied Sciences in Konin, Konin, Poland; 4 The President Stanislaw Wojciechowski Calisia University, Department of Computer Science, Polytechnic Faculty, University of Kalisz, Kalisz, Poland; Khalifa University, UNITED ARAB EMIRATES

## Abstract

**Background:**

Diabetes remains a major public health concern in the United States, with a complex interplay of behavioral, demographic, and clinical risk factors. This study aims to identify the three best-performing machine learning models for diabetes risk prediction and to visualize the most influential predictors affecting diabetes likelihood. By leveraging a large, representative dataset, the study contributes to evidence-based strategies for targeted prevention.

**Methods:**

Data were obtained from the 2015 Behavioral Risk Factor Surveillance System (BRFSS), a nationally representative, population-based survey collecting information on health behaviors, chronic conditions, and preventive care. The analytical sample included 253,680 adult respondents and over twenty features encompassing sociodemographic variables (e.g., age, sex, race, income, education), health behaviors (e.g., smoking, physical activity, diet), and outcomes (e.g., BMI, hypertension, diabetes status). Eighteen machine learning models were trained and evaluated, including AdaBoost, Extra Trees Classifier, C5.0 Decision Tree, and CatBoost. Models were assessed using predictive accuracy and AUC scores. SHAP (SHapley Additive exPlanations) analysis was used to interpret the top model and examine how changes in key features influence diabetes risk.

**Results:**

Among the evaluated models, the Extra Trees Classifier achieved the highest predictive accuracy (>90%) and an AUC of 0.99. AdaBoost and CatBoost also demonstrated strong performance. Feature importance analysis identified BMI, age, general health status, income, physical health days, and education as the top predictors. A nonlinear association between income and diabetes risk was observed, with the highest prevalence in individuals earning $20,000–$25,000. Risk was also elevated in individuals aged 65–69 and those reporting poor general health. Hypertension showed a strong positive correlation with diabetes risk.

**Conclusions:**

Machine learning models, particularly tree-based ensemble methods, offer robust tools for diabetes risk prediction. These findings support their integration into public health analytics for personalized risk assessment and data-driven prevention strategies.

## Introduction

Diabetes is a chronic metabolic disease characterized by hyperglycemia, resulting from defects in insulin secretion, insulin action or both [1]. The disease is becoming an increasingly serious public health problem worldwide, affecting millions of people and generating significant costs for health systems. While genetic factors play a role in the development of diabetes, non-medical determinants such as lifestyle and socioeconomic conditions are increasingly recognized as key factors influencing the incidence and progression of the disease. However, heart disease is often comorbid with diabetes and can increase the risk of developing diabetes, as well as exacerbate its course [2,3]. Understanding these determinants is essential for developing effective prevention and intervention strategies, as well as for reducing the economic burden of diabetes.

Among the most important non-medical determinants of diabetes are:

- Lifestyle, which consists of many factors, such as: High body mass index (BMI), which is a strong predictor of type 2 diabetes. Obesity and overweight increase the risk of insulin resistance, which is a key factor in the development of this form of diabetes [2,4,5].
- Low levels of physical activity are another important risk factor for type 2 diabetes. Regular physical activity improves insulin sensitivity and helps maintain a healthy body weight, reducing the risk of developing the disease [6–8].
- Age is a significant factor influencing the occurrence of type 2 diabetes; however, this relationship is nonlinear and depends on multiple interrelated variables [9], including diet, BMI, lifestyle, and socioeconomic determinants. Epidemiological data indicate that the proportion of diet-related type 2 diabetes cases is higher among younger individuals (20–25 years) and declines with age. Although younger individuals are more susceptible to developing diabetes due to dietary factors, the highest absolute incidence of type 2 diabetes is observed in the 45–60 age group, which is attributed to the cumulative effect of metabolic risk factors, such as lifestyle choices, diet, and BMI, during this stage of life.
- Furthermore, an elevated BMI is strongly associated with an increased risk of diabetes, regardless of age. However, age plays a crucial role in both the onset and progression of type 2 diabetes and its associated complications [10]. The prevalence of type 2 diabetes increases with advancing age, primarily due to the progressive decline in pancreatic β-cell function and the exacerbation of insulin resistance.
- Income is also an important factor [11,12], lower income is closely associated with a higher risk of developing type 2 diabetes. In lower income groups, a higher incidence of other diseases was also observed: hypertension, overweight/obesity and lower levels of physical activity, which additionally increase the risk of developing diabetes. The influence of income results from, among others, limited access to healthier food, sports and recreational facilities and paid health care.
- Smoking is associated with an increased risk of type 2 diabetes and cardiovascular complications in people with diabetes [13,14].

From a health economics perspective, diabetes is a significant burden on society. The costs associated with diabetes treatment, lost productivity and premature mortality are enormous [15]. Effective strategies for prevention and early detection of diabetes, targeting the modification of non-medical determinants, can yield significant economic benefits.

The objective of this article is to compare various machine learning methods—such as AdaBoost Classifier, Extra Trees Classifier, C5.0 Decision Tree, CatBoost, and a range of other commonly used models including Decision Tree Classifier, Random Forest Classifier, K-Nearest Neighbors (KNN), Histogram-based Gradient Boosting, LightGBM Classifier, XGBoost Classifier, Gradient Boosting Classifier, Ridge Classifier, Linear Discriminant Analysis, Logistic Regression, Quadratic Discriminant Analysis, Naive Bayes Classifier, Nearest Centroid Classifier, and Isolation Forest—in predicting the risk of diabetes. The analysis will based on over 250,000 observations from the 2015 Behavioral Risk Factor Surveillance System (BRFSS), a health-related telephone survey conducted annually by the Centers for Disease Control and Prevention (CDC). The study aims to identify and recommend the three best-performing techniques based on predictive accuracy. Additionally, the article will visualize the top determinants of diabetes risk for the best predictive model, demonstrating how changes in these factors influence the likelihood of diabetes. This comprehensive approach seeks to provide actionable insights into diabetes prevention and risk assessment. Based on the best-performing model identified in this study, an web-application will be developed to enable patients and healthcare professionals to better estimate diabetes risk, facilitating more informed decision-making and personalized prevention strategies.

Diabetes remains a critical public health issue in the United States, with increasing interest in leveraging machine learning to improve risk prediction and prevention strategies. This study advances current research by combining robust predictive modeling with interpretability techniques to better understand the key drivers of diabetes risk. The main contributions of this paper are as follows:

- This study systematically evaluates and compares 18 machine learning models to identify the top three algorithms for accurate diabetes risk prediction using a large, nationally representative dataset.
- It applies SHAP analysis to enhance model interpretability and visualize how key features influence diabetes likelihood.
- The findings contribute to public health analytics by demonstrating the value of tree-based ensemble models in enabling personalized risk assessments and informing data-driven prevention strategies.

The structure of the article follows a clear and logical framework appropriate for empirical research in health economics. The Introduction outlines the key determinants of diabetes and clearly states the research objective. The Methods section describes the dataset in detail, along with the applied machine learning techniques, model evaluation metrics, and visualization methods. In the Results section, findings from the analysis are presented, comparing the performance of various machine learning models and examining how major risk factors relate to diabetes probability. The Discussion section interprets these findings in the context of existing literature, discusses the study’s limitations, and proposes directions for future research. Finally, the article concludes by summarizing the key results and their implications for data-driven diabetes risk assessment.

## Materials and methods

In this section, the dataset will be introduced, followed by an overview of the research methods employed, including machine learning techniques, visualization of results, and the evaluation of classification model performance. Finally, the research design will be presented in a diagram to provide a structured overview of the analytical approach.

### Data source

The BRFSS is a comprehensive, population-based survey that collects data on health-related risk behaviors, chronic health conditions, and the use of preventive services among adults in the United States. This dataset comprises 253,680 of observations and includes more than twenty features that provide insights into various determinants of health, including diabetes. Key variables in the dataset encompass demographic information (age, sex, race, and education), health behaviors (such as smoking status, physical activity, and dietary habits), and health outcomes (including self-reported diabetes status, body mass index, and hypertension) [16]. The BRFSS employs a random-digit-dialing methodology, ensuring a representative sample of the adult population, although it is important to note that some variables are self-reported, which may introduce biases such as social desirability or recall bias [17,18]. The dataset’s extensive scope allows for the exploration of correlations between socioeconomic factors and health outcomes, making it a valuable resource for understanding the multifaceted nature of diabetes determinants. By analyzing these variables, researchers can identify high-risk populations and inform public health strategies aimed at reducing the incidence of diabetes and improving health outcomes across diverse communities. The BRFSS serves as a critical tool for policymakers and health professionals to monitor trends in health behaviors and outcomes, ultimately guiding interventions to address the growing diabetes epidemic in the United States [19]. The study utilized the 2015 Behavioral Risk Factor Surveillance System (BRFSS) dataset, which is publicly available and de-identified. As such, analyses of BRFSS data are exempt from Institutional Review Board (IRB) review at the Centers for Disease Control and Prevention (CDC) under 45 CFR 46.101(b)(2), as confirmed in other CDC publications [20].

The dataset comprises a sample of 253,680 respondents, providing a comprehensive representation of the surveyed population in the USA. Table 1 shows that males account for 44% of the sample, and the average age category falls within the 55–59 age range, reflecting a middle-aged to older demographic. The educational attainment of respondents, measured on a categorical scale, averages 5, corresponding to at least some college or technical school education. Similarly, the average income category is 6, indicating a household income of at least $35,000 per year, which suggests a moderate socioeconomic profile. The prevalence of key health conditions varies significantly, with 14% of respondents reporting diabetes or prediabetes, 43% diagnosed with high blood pressure, and 42% exhibiting elevated cholesterol levels, while 96% had undergone a cholesterol check in the past five years. A detailed definition and the levels of all variables are provided in S1 File, while the histograms of all variables included in the analysis are presented in S1 Fig in the supplementary materials.

**Table 1:** Descriptive statistics of BRFSS dataset.

Variable	Mean	Standard Deviation	Minimum	25%	Median	75%	Maximum
Diabetes_binary	0,14	0,35	0	0	0	0	1
HighBP	0,43	0,49	0	0	0	1	1
HighChol	0,42	0,49	0	0	0	1	1
CholCheck	0,96	0,19	0	1	1	1	1
BMI	28,38	6,61	12	24	27	31	98
Smoker	0,44	0,50	0	0	0	1	1
Stroke	0,04	0,20	0	0	0	0	1
HeartDiseaseorAttack	0,09	0,29	0	0	0	0	1
PhysActivity	0,76	0,43	0	1	1	1	1
Fruits	0,63	0,48	0	0	1	1	1
Veggies	0,81	0,39	0	1	1	1	1
HvyAlcoholConsump	0,06	0,23	0	0	0	0	1
AnyHealthcare	0,95	0,22	0	1	1	1	1
NoDocbcCost	0,08	0,28	0	0	0	0	1
GenHlth	2,51	1,07	1	2	2	3	5
MentHlth	3,18	7,41	0	0	0	2	30
PhysHlth	4,24	8,72	0	0	0	3	30
DiffWalk	0,17	0,37	0	0	0	0	1
Sex	0,44	0,50	0	0	0	1	1
Age	8,03	3,05	1	6	8	10	13
Education	5,05	0,99	1	4	5	6	6
Income	6,05	2,07	1	5	7	8	8

Note: 253 680 observations.

Source: own elaboration.

In terms of lifestyle and behavioral factors, the sample exhibits notable patterns. Table 1 presents mean BMI above 28 kg/m2, indicating a predominantly overweight or obese population. Past or current smokers constitute 44% of respondents, while 4% reported a history of stroke and 9% have been diagnosed with coronary heart disease or experienced a heart attack. Despite these health challenges, 76% of participants engaged in physical activity within the last 30 days (excluding occupational activities). Dietary habits reveal that 63% consume fruit daily, while 81% consume vegetables daily, suggesting a relatively health-conscious dietary pattern. However, 6% of respondents reported heavy alcohol consumption, defined as ≥14 drinks per week for men and ≥7 drinks per week for women. Access to healthcare appears relatively high, with 95% of respondents covered by health insurance, though 8% indicated they had foregone a doctor’s visit in the past year due to cost-related barriers.

Self-reported health status presents a nuanced perspective on overall well-being. On a general health assessment scale from 1 (excellent) to 5 (poor), the mean score is 2.51, situating respondents’ perceived health between good and very good. Mental and physical health burdens are evident, with respondents reporting an average of 3.18 days of poor mental health and 4.24 days of poor physical health in the past 30 days. Mobility issues affect a significant minority, with 17% experiencing difficulty walking or climbing stairs, underscoring the presence of functional limitations within the surveyed population. The correlation matrix of the variables is provided in S2 Fig in the supplementary materials. S2 Fig indicates that the strongest correlations are observed between variables related to physical health and mobility issues, as well as between income and education.

## Methods

In this subsection, all machine learning techniques used for selecting the best predictive model will be presented. Additionally, the model evaluation method will be discussed to determine the most accurate predictive model.

### Machine learning

In this study, we employed a variety of machine learning methods to analyze the determinants of diabetes, focusing on their predictive capabilities and comparative performance. The methods selected for this analysis include both classical and ensemble techniques: AdaBoost Classifier, Extra Trees Classifier, C5.0 Decision Tree, CatBoost, Decision Tree Classifier, Gradient Boosting Classifier, Histogram-based Gradient Boosting, Isolation Forest, K-Nearest Neighbors (KNN), LightGBM Classifier, Linear Discriminant Analysis (LDA), Logistic Regression, Ridge Classifier, Naive Bayes Classifier, Nearest Centroid Classifier, Quadratic Discriminant Analysis (QDA), Random Forest Classifier, and XGBoost Classifier. Each of these methods offers unique advantages and is suitable for different aspects of diabetes prediction and classification.

The AdaBoost Classifier, known for its ability to improve the accuracy of weak classifiers, was utilized to enhance the performance of decision trees by combining multiple weak learners into a strong learner [21,22]. Similarly, the Extra Trees Classifier, which builds multiple decision trees and merges them to obtain a more accurate and stable prediction, was included for its robustness against overfitting [21]. The C5.0 Decision Tree algorithm is an advanced version of the traditional decision tree, providing better accuracy and efficiency through boosting techniques [21]. In contrast, the CatBoost algorithm, which is particularly effective with categorical features, was employed to handle the diverse data types typically found in diabetes datasets [21,23].

Gradient Boosting Classifier and its histogram-based variant have gained popularity due to their efficiency and high predictive performance in large datasets. These methods sequentially build trees, where each new tree corrects the errors of the previous ones, making them particularly effective for complex datasets [15]. Isolation Forest, a method specifically designed for anomaly detection, was included to identify outliers in diabetes data, which can significantly affect model performance [21]. K-Nearest Neighbors (KNN) was also utilized for its simplicity and effectiveness in classification tasks, particularly when the dataset is small and the decision boundary is irregular [21].

LightGBM Classifier and XGBoost Classifier are both gradient boosting frameworks that have shown exceptional performance in various machine learning competitions. LightGBM is optimized for speed and efficiency, making it suitable for large datasets, while XGBoost is known for its scalability and performance [24]. Linear Discriminant Analysis (LDA) and its variant, Quadratic Discriminant Analysis (QDA), were included for their effectiveness in dimensionality reduction and classification, particularly in cases where the classes are normally distributed [25,26]. Logistic Regression and Ridge Classifier were also employed as baseline models due to their interpretability and effectiveness in binary classification tasks [21].

Naive Bayes Classifier and Nearest Centroid Classifier were included for their simplicity and effectiveness in handling high-dimensional data, particularly in scenarios where the independence assumption holds [21]. Each of these classifiers was evaluated based on their predictive accuracy, sensitivity, specificity, and computational efficiency. The performance metrics were compared using cross-validation techniques to ensure the robustness of the results.

The overall methodology involved preprocessing the diabetes dataset to handle missing values and outliers, followed by feature selection to identify the most relevant predictors. Each machine learning model was trained and tested using a stratified k-fold cross-validation approach to ensure that the results were generalizable and not biased by the specific data split. The comparative analysis of these methods provides insights into their relative strengths and weaknesses in predicting diabetes, ultimately contributing to the understanding of diabetes determinants and enhancing predictive accuracy in clinical settings.

### Visualization

The utilization of Shapley Additive Explanations (SHAP) values in the context of predicting diabetes risk has emerged as a powerful tool for visualizing the impact of various determinants on the probability of developing diabetes. SHAP values provide a method to interpret complex machine learning models, particularly those based on eXtreme Gradient Boosting (XGBoost), by quantifying the contribution of each feature to the model’s predictions. This approach not only enhances the interpretability of the model but also allows for a nuanced understanding of how individual factors, such as age, body mass index, and lifestyle choices, influence diabetes risk [27,28]. By employing SHAP values, researchers can generate visualizations such as summary plots and decision plots, which elucidate the positive or negative impacts of specific features on diabetes probability [28,29]. For instance, a SHAP summary plot can reveal the average effect of each feature across the dataset, while decision plots can illustrate how changes in feature values affect individual predictions [28]. This level of interpretability is crucial for healthcare practitioners, as it facilitates informed decision-making and personalized interventions for diabetes prevention [30,31]. Overall, the integration of SHAP values in diabetes risk modeling underscores the importance of explainable AI in medical applications, enhancing both the accuracy and transparency of predictive analytics [32,33].

### Evaluation of classification model performance

The evaluation of classification model performance in the context of diabetes determinants is critical for understanding the effectiveness of predictive analytics in healthcare. Key metrics such as precision, sensitivity, specificity, F1-score, accuracy, area under the curve (AUC), R², and mean squared error (MSE) derived from the confusion matrix provide a comprehensive overview of model efficacy. Precision, defined as the ratio of true positives to the sum of true and false positives, is crucial for assessing the reliability of positive predictions, particularly in imbalanced datasets common in diabetes research [34]. Sensitivity (or recall) measures the proportion of actual positives correctly identified, while specificity assesses the true negative rate, both of which are essential for understanding the model’s performance in clinical settings [34]. The F1-score, which harmonizes precision and sensitivity, is particularly useful when the class distribution is uneven, as it provides a single metric that balances both concerns [34].

Accuracy, representing the overall correctness of the model, can be misleading in cases of class imbalance, thus necessitating the use of AUC, which evaluates the model’s ability to distinguish between classes across various threshold settings [35]. R² provides insight into the proportion of variance explained by the model, while MSE quantifies the average squared difference between predicted and actual values, offering a measure of predictive accuracy [36]. integration of these metrics allows for a nuanced understanding of model performance, enabling researchers to make informed decisions about the applicability of their models in real-world scenarios. In diabetes research, where the stakes are high, employing a robust evaluation framework is essential for developing reliable predictive tools that can enhance patient outcomes and inform clinical practices [37].

### Research design

**Fig 1:**
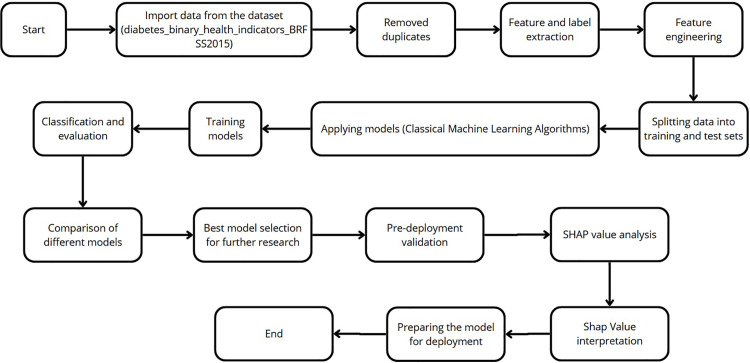
Research framework.

Fig 1 illustrates the research design and the sequential steps undertaken to develop the study. First, the Behavioral Risk Factor Surveillance System dataset was obtained from the official Kaggle website. Next, data preprocessing was performed, leveraging a literature review on diabetes determinants to guide feature selection. To address the issue of uneven data distribution, various resampling techniques were applied, including random oversampling (ROS) and synthetic over-sampling techniques (SMOTE, ADASYN) [38,39].

Subsequently, 18 machine learning models were constructed and evaluated, including AdaBoost Classifier, Extra Trees Classifier, C5.0 Decision Tree, CatBoost, Decision Tree Classifier, Gradient Boosting Classifier, Histogram-based Gradient Boosting, Isolation Forest, K-Nearest Neighbors (KNN), LightGBM Classifier, Linear Discriminant Analysis (LDA), Logistic Regression, Ridge Classifier, Naive Bayes Classifier, Nearest Centroid Classifier, Quadratic Discriminant Analysis (QDA), Random Forest Classifier, and XGBoost Classifier. Each model’s performance was systematically compared, with the dataset split into training and testing sets in an 80:20 ratio. Finally, the SHAP framework was employed to interpret the predictive results, ensuring transparency in model decision-making. The entire process was implemented in Python, and the technical workflow of the study is depicted in Fig 1.

## Results

In this section, the following results summarize the comparative performance of the evaluated machine learning algorithms and provide insights into the most influential predictors of diabetes based on feature importance analysis.

The descriptive statistics of the dataset, as presented in Table 1, indicate a pronounced imbalance between diabetes cases and non-cases, with the prevalence of diabetes constituting approximately 14% of the sample. This substantial class imbalance introduces potential challenges in model training, as classifiers may be biased toward predicting the majority class, thereby diminishing sensitivity to the minority class. Measures of central tendency and dispersion further reveal that individuals with diabetes exhibit distinct characteristics in key demographic and clinical variables, such as age, BMI, and fasting glucose levels, suggesting potential predictive features for classification models.

**Fig 2:**
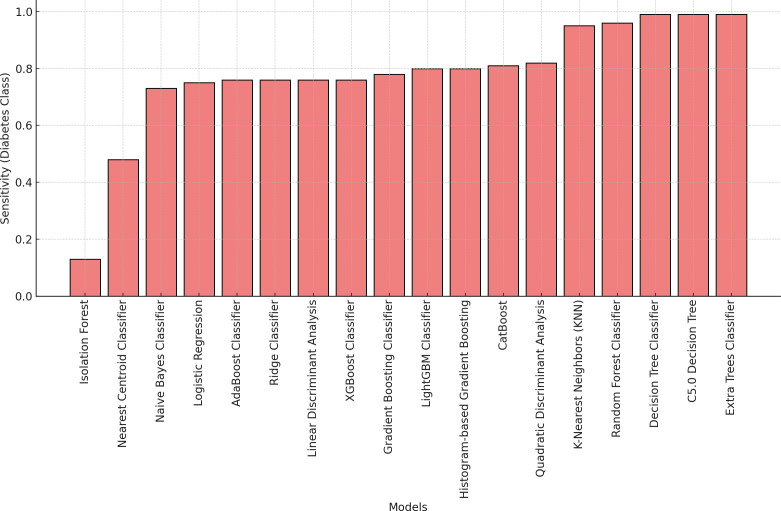
Model sensitivity comparison for diabetes classification models.

**Fig 3:**
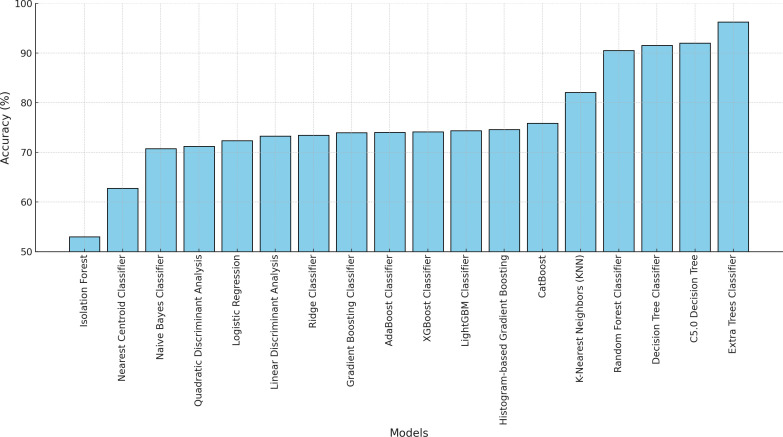
Model accuracy comparison for diabetes classification models.

**Fig 4:**
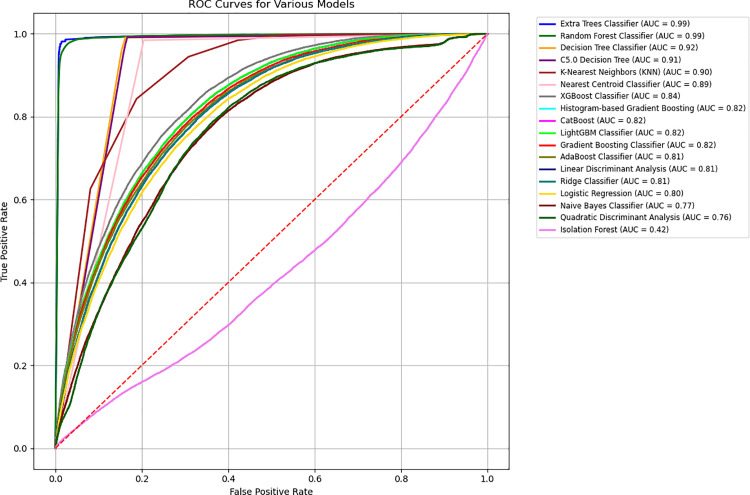
ROC curves for diabetes classification models.

Figs 2 and 3 illustrate the predictive performance of 18 classification models based on accuracy and sensitivity, demonstrating substantial variability in their effectiveness. The Extra Trees Classifier consistently outperforms other models, achieving accuracy and sensitivity levels exceeding 90%, indicating its robustness in distinguishing between diabetes cases and non-cases. Additionally, as shown in Fig 4, the Receiver Operating Characteristic (ROC) curves for all predictive models further confirm the superior performance of the Extra Trees Classifier, with an AUC of 0.99, highlighting its strong discriminatory power. In the context of imbalanced data, accuracy and sensitivity provide insights into correct classifications and the ability to detect minority class instances, while the ROC curve and AUC measure overall model performance across varying classification thresholds, ensuring that predictive capabilities are not skewed by class distribution disparities.

Table 2 provides a comparative assessment of predictive model performance, highlighting the superior efficacy of the Extra Trees Classifier. This model achieves an accuracy exceeding 96%, alongside a precision of 94% and a recall rate of 99%, indicating strong predictive capability across performance metrics. The AUC surpasses 96%, demonstrating robust discrimination between diabetes cases and non-cases. The C5.0 Decision Tree emerges as the second-best classifier, with an accuracy of 92% and comparable precision and recall rates, followed closely by the Decision Tree Classifier, which exhibits similar predictive strength. These findings underscore the potential of ensemble-based and decision tree-based algorithms in diabetes classification (more details in S3 Fig).

**Table 2:** Model performance metrics.

Model	Accuracy (%)	Precision	Recall	AUC/ R²/ MSE
Extra Trees Classifier	0.96	0.94	0.99	0.96
C5.0 Decision Tree	0.92	0.99	0.99	0.92
Decision Tree Classifier	0.92	0.99	0.99	0.92
Random Forest Classifier	0.90	0.87	0.96	0.90
K-Nearest Neighbors (KNN)	0.82	0.76	0.95	0.82
CatBoost	0.76	0.81	0.81	0.76
Histogram-based Gradient Boosting	0.75	0.73	0.8	0.75
LightGBM Classifier	0.74	0.72	0.80	0.75
XGBoost Classifier	0.74	0.72	0.76	0.73
AdaBoost Classifier	0.74	0.76	0.76	0.74
Gradient Boosting Classifier	0.74	0.72	0.78	0.74
Ridge Classifier	0.73	0.72	0.76	0.73
Linear Discriminant Analysis	0.73	0.72	0.76	0.73
Logistic Regression	0.72	0.72	0.75	0.73
Quadratic Discriminant Analysis	0.71	0.68	0.82	0.71
Naive Bayes Classifier	0.71	0.70	0.73	0.71
Nearest Centroid Classifier	0.63	0.68	0.48	0.63
Isolation Forest	0.53	0.65	0.13	0.53

Conversely, models such as the Nearest Centroid Classifier and Isolation Forest exhibit markedly lower predictive power, with accuracy, precision, and AUC values falling below 70%. The diminished performance of these models suggests that linear or distance-based classification approaches may be less suitable for the dataset’s underlying structure. Given the dataset’s imbalanced nature, model performance should be interpreted in the context of recall and AUC metrics, as high accuracy alone may not adequately capture predictive reliability. The findings reinforce the importance of algorithm selection in optimizing diabetes prediction and highlight the need for careful consideration of dataset characteristics in model evaluation.

**Fig 5:**
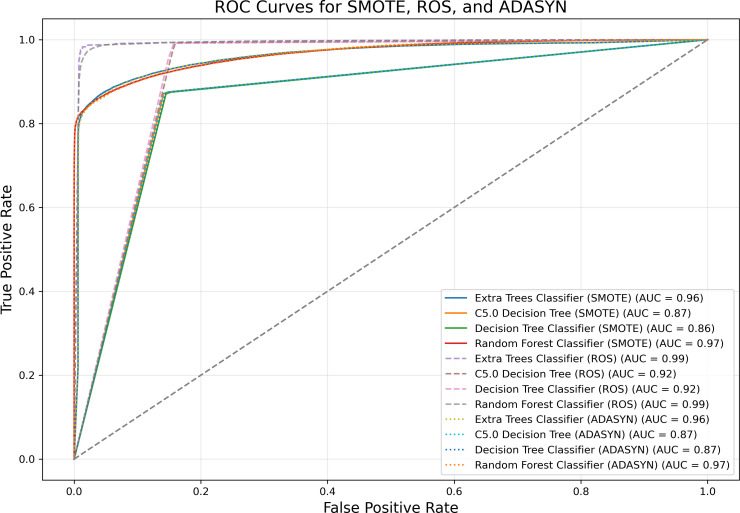
Comparison of three techniques of oversampling for three best models.

Fig 5 illustrates the application of three oversampling techniques—Random Over-Sampling (ROS), Synthetic Minority Over-sampling Technique (SMOTE), and Adaptive Synthetic Sampling (ADASYN)—for the three best-performing predictive models of diabetes. The results indicate that ROS yielded the highest performance across all models. According to the data presented in S1 Table, the ROS technique demonstrated superior performance when applied to the Extra Trees Classifier, yielding the highest values across key evaluation metrics, including accuracy, recall, and AUC. Based on this observation, further calculations were conducted using the Extra Trees Classifier to enhance predictive accuracy and model robustness.

**Fig 6:**
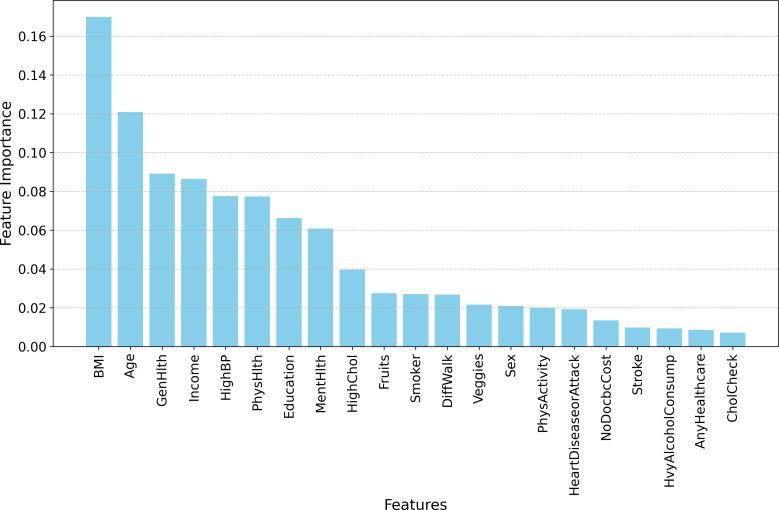
Feature importance ranking for Extra Trees Classifier.

Fig 6 highlights the key determinants influencing the predictive performance of the model, with variables such as body mass index (BMI), age, general health status (GenHlth), income level, physical health (PhysHlth), and education emerging as the most significant contributors. These features exhibit a strong positive association with diabetes classification, indicating their relevance in both identifying at-risk individuals and informing preventive strategies. The prominence of BMI and age aligns with established medical and economic literature, where higher BMI and older age are consistently linked to an increased likelihood of diabetes onset. Similarly, socioeconomic factors, including income and education, play a crucial role, as lower-income individuals and those with limited education may face barriers to healthcare access, preventive care, and healthier lifestyles, exacerbating diabetes risk. The observed importance of these features underscores the multidimensional nature of diabetes prediction, integrating clinical, behavioral, and socioeconomic determinants.

**Fig 7:**
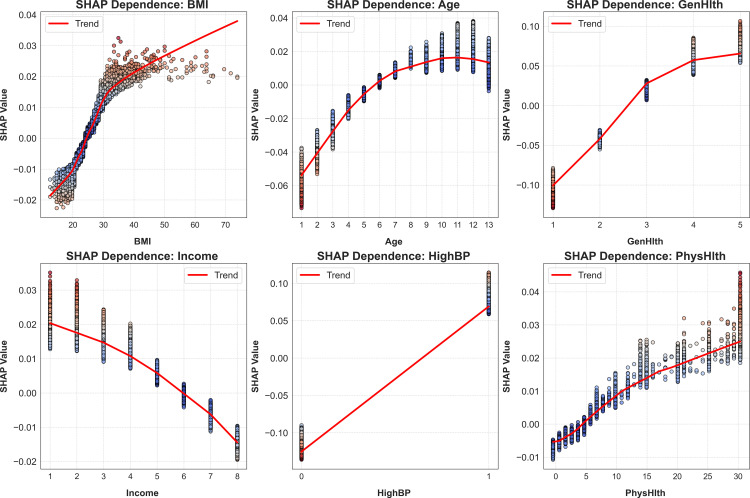
Dependency of diabetes with top 6 diabetes determinants with SHAP values.

Fig 7 illustrates the impact of the six most significant determinants of diabetes risk, highlighting distinct patterns across different predictor variables. With respect to BMI, the risk of diabetes increases most sharply for individuals with a BMI between 25 and 30, aligning with existing evidence on the heightened susceptibility of overweight and moderately obese individuals. Age-related risk follows a nonlinear trajectory, with diabetes prevalence rising markedly from the sixth age category (>45 years) and peaking in the tenth category (65–69 years), consistent with metabolic and physiological changes associated with aging. Similarly, self-reported general health status exhibits a strong negative association with diabetes risk, with individuals who classify their health as fair (category 4) or poor (category 5) demonstrating the highest probability of developing the condition.

Economic and health behavior factors further reveal complex relationships with diabetes risk. Household income follows an inverted U-shaped pattern, with the highest risk observed among individuals earning $20,000–$25,000 annually. Risk increases across the first four income brackets (up to $25,000) before gradually declining in higher-income groups, suggesting a potential link between financial constraints, healthcare access, and health outcomes. Interestingly, the number of days with poor physical health in the last 30 days exhibits a counterintuitive trend, where a higher reported frequency of poor physical health days is associated with a lower likelihood of diabetes, potentially reflecting reverse causality or variations in healthcare-seeking behavior. In contrast, high blood pressure demonstrates a strong positive correlation with diabetes risk, reinforcing the well-documented interplay between hypertension and metabolic disorders. These findings underscore the multifaceted nature of diabetes determinants and highlight the need for targeted interventions addressing both clinical and socioeconomic risk factors.

## Discussion

This section interprets the key findings from the analysis, placing them in the context of existing literature and discussing their implications for diabetes risk prediction and public health strategies. The increasing prevalence of diabetes, particularly type 2 diabetes, necessitates the development of robust predictive models that can effectively identify individuals at risk. Recent advancements in AI particularly in machine learning (ML) have shown promise in enhancing the accuracy of diabetes risk prediction. Few systematic reviews highlight various ML and deep learning models that have been employed to predict type 2 diabetes, emphasizing the potential of these technologies in clinical settings [1,24,40].

In a comparative analysis utilizing data from the BRFSS, the Extra Trees Classifier emerged as the most effective model, achieving an accuracy exceeding 90% and an AUC of 0.99, thereby demonstrating its superior discriminatory power in distinguishing between diabetes cases and non-cases [21].

The analysis of key determinants influencing diabetes risk reveals several significant factors, including BMI, age, general health status, income level, physical health, and education. Specifically, individuals with a BMI between 25 and 35 exhibit a markedly increased risk of diabetes, which is confirmed with findings from Hu et al. that link elevated BMI to heightened diabetes susceptibility [6,18]. Age-related risk follows a nonlinear trajectory, with a pronounced increase in diabetes prevalence observed in individuals over 45 years, aligning with the metabolic and physiological changes associated with aging [19]. Furthermore, self-reported general health status is inversely correlated with diabetes risk; individuals rating their health as fair or poor demonstrate a significantly higher likelihood of developing diabetes, echoing the findings of Graham et al. regarding the health characteristics of older adults with prediabetes [4].

Economic factors also play a crucial role in diabetes risk stratification. The analysis indicates an inverted U-shaped relationship between household income and diabetes risk, with the highest risk observed among individuals earning between $20,000 and $25,000 annually. This pattern suggests that financial constraints may limit access to healthcare resources, thereby exacerbating health outcomes [21]. Interestingly, the relationship between reported days of poor physical health and diabetes risk appears counterintuitive; higher frequencies of poor physical health days correlate with a lower likelihood of diabetes, potentially reflecting reverse causality or variations in healthcare-seeking behavior [21]. Additionally, the strong positive correlation between high blood pressure and diabetes risk reinforces the established interplay between hypertension and metabolic disorders, underscoring the need for integrated approaches to manage these interrelated health issues.

AI algorithms including machine learning models, particularly those employing advanced techniques such as SHAP values, offer valuable insights into the predictive performance of diabetes risk models. SHAP values facilitate the interpretation of model predictions by quantifying the contribution of each feature to the overall prediction, thereby enhancing the transparency and usability of these models in clinical practice (Ergün, 2023). This interpretability is crucial for healthcare providers as it allows for tailored interventions based on individual risk profiles. Moreover, the integration of various machine learning classifiers, as demonstrated in studies by Maniruzzaman et al., can further optimize risk stratification by addressing challenges related to missing values and outliers, ultimately improving the accuracy of diabetes predictions [21].

In conclusion, the application of machine learning methodologies in diabetes risk prediction represents a significant advancement in public health research. The Extra Trees Classifier’s outstanding performance, coupled with the identification of critical risk factors, underscores the potential for these models to inform targeted interventions and improve health outcomes. As the landscape of diabetes continues to evolve, ongoing research and refinement of predictive models will be essential in addressing the multifaceted determinants of this chronic disease.

Depending on the type, format, and characteristics of the data, various machine learning techniques, deep learning approaches, and artificial neural networks are employed [41–43]. For tabular data with well-defined features, decision trees, Random Forest, and other tree-based methods—classical machine learning techniques—tend to perform effectively. Conversely, for large and diverse datasets, such as images, text, or sequences, deep learning techniques are generally more suitable. In the broader context of diabetes diagnosis, both machine learning and deep learning methods have been applied [44–46]. In our study, the Extra Trees Classifier emerged as the most effective technique. The conducted computations indicate significant differences in accuracy, precision, and recall among models, with Extra Trees Classifier and Random Forest demonstrating the highest accuracy for the given dataset. When developing AI models, it is crucial to experiment with a variety of machine learning and deep learning techniques to select the most appropriate method.

### Limitations

The current research presents several limitations that warrant consideration. Firstly, the reliance on data from the BRFSS may introduce biases due to self-reported measures, which can be influenced by social desirability and recall bias. This limitation is particularly relevant when assessing health behaviors and socioeconomic factors, as individuals may underreport or overreport their conditions and experiences. Additionally, while the Extra Trees Classifier demonstrated superior predictive accuracy, the study’s cross-sectional design limits the ability to establish causal relationships between the identified risk factors and diabetes. Panel data are not accounted for, which may obscure the understanding of how these risk factors interact over time and contribute to the onset of diabetes. Furthermore, the study’s focus on a specific population in the United States may limit the generalizability of the findings to other regions or demographic groups, potentially overlooking variations in risk factors across different populations.

Future research directions should focus on addressing these limitations and expanding the understanding of diabetes risk factors. One potential avenue is the longitudinal analysis of diabetes risk, which would allow researchers to observe changes over time and establish causal relationships between risk factors and diabetes onset. This approach could provide valuable insights into the progression of diabetes risk and the effectiveness of interventions over time. Additionally, incorporating qualitative methods could provide deeper insights into the lived experiences of individuals at risk for diabetes, particularly regarding their health behaviors and socioeconomic challenges. Future work could focus on expanding the dataset to include a wider range of demographic groups and diverse health data beyond current determinants, enhancing the model’s generalizability and robustness [47,48]. Subsequent studies could investigate advanced ensemble learning methods or novel hybrid models, combining the strengths of multiple classifiers to potentially achieve even higher predictive accuracy and better handle the complexity of diabetes risk determinants [49]. Understanding the context in which individuals make health-related decisions can inform more tailored and effective intervention strategies.

## Conclusions

This study systematically evaluates the predictive accuracy of various machine learning models in estimating diabetes risk using data from the 2015 BRFSS Among the 18 models assessed, the Extra Trees Classifier consistently demonstrated superior predictive power, achieving the highest accuracy, sensitivity, and AUC scores. This highlights its effectiveness in distinguishing between individuals with and without diabetes. The study also underscores the importance of integrating advanced machine learning techniques into public health research, as these methods can provide valuable insights for early detection and risk stratification. By leveraging a large-scale dataset of over 250,000 observations, this research reinforces the applicability of data-driven approaches in enhancing clinical decision-making and public health interventions.

The findings also emphasize the multifaceted nature of diabetes risk, where both clinical and socioeconomic factors play crucial roles. BMI, age, and general health status emerged as the strongest predictors, with diabetes risk peaking for individuals aged 65–69 years and those classified as having poor general health. Additionally, the study identifies an inverted U-shaped relationship between income and diabetes risk, with individuals in the $20,000–$25,000 bracket facing the highest probability of developing the disease. Interestingly, the study also highlights a counterintuitive trend regarding the number of poor physical health days, suggesting potential reverse causality or behavioral factors influencing self-reported health data. These insights are critical for policymakers and healthcare practitioners, as they underscore the necessity for targeted preventive strategies addressing both medical and socioeconomic determinants. The integration of these findings into clinical decision support systems could enhance personalized risk assessments and facilitate more effective diabetes prevention and management programs.

## Supporting information

S1 File Coding of variables. (DOCX)

S1 Fig Histograms of variables. (PNG)

S2 Fig Correlation matrix between variables. (PNG)

S3 Fig Confusion matrix for all models. (PNG)

S1 Table Comparison of effectiveness of different techniques for unbalanced dataset. (DOCX)

## Abbreviations

ML: machine learning
AI: artificial intelligence
BRFSS: Behavioral Risk Factor Surveillance System
AUC: Area Under the Curve
BMI: Body Mass Index
SHAP values: Shapley Additive Explanations
XGBoost: Extreme Gradient Boosting
ADASYN: Adaptive Synthetic Sampling
SMOTE: Synthetic Minority Over-sampling Technique
ROS: Random OverSampling
ROC: Receiver Operating Characteristic
