# Supplementary material for: AI-driven analysis of diabetes risk determinants in U.S. adults: Exploring disease prevalence and health factors
Source: PLoS One. 2025 Sep 3;20(9):e0328655. doi: 10.1371/journal.pone.0328655 (PMC12407459; doi:10.1371/journal.pone.0328655)
Supplement: S1 File — (DOCX) [file pone.0328655.s001.docx]

**S1 File. Coding of variables.**

1. Diabetes_binary - Diabetes status. (Data displayed during model prediction / not to be entered by the user/patient)
   • 0 = No diabetes (type 2)
   • 1 = Diabetes (type 2) or prediabetes
2. HighBP - High blood pressure (declaration)
   • 0 = No high blood pressure
   • 1 = High blood pressure
3. HighChol- High cholesterol level (declaration)
   • 0 = No high cholesterol
   • 1 = High cholesterol
4. CholCheck - Cholesterol check in the past 5 years.
   • 0 = No check
   • 1 = Check performed
5. BMI - Body Mass Index (BMI) — numeric value.
6. Smoker - Smoking (at least 100 cigarettes in a lifetime).
   • 0 = Never smoked
   • 1 = Past or current smoker
7. Stroke.
   • 0 = Never had a stroke
   • 1 = Diagnosed stroke
8. HeartDiseaseorAttack - Coronary heart disease or heart attack.
   • 0 = Never diagnosed
   • 1 = Diagnosed coronary heart disease or heart attack
9. PhysActivity- Physical activity in the last 30 days (excluding professional work).
   • 0 = No physical activity
   • 1 = Physical activity undertaken
10. Fruits- Daily consumption of fruits.
    • 0 = No
    • 1 = Yes
11. Veggies- Daily consumption of vegetables.
    • 0 = No
    • 1 = Yes
12. HvyAlcoholConsump - Heavy alcohol consumption (men ≥14 drinks/week, women ≥7 drinks/week).
    • 0 = No
    • 1 = Yes
13. AnyHealthcare- Having health insurance.
    • 0 = No insurance
    • 1 = Has insurance
14. NoDocbcCost- No doctor's visit due to costs in the last 12 months.
    • 0 = No
    • 1 = Yes
15. GenHlth- General health assessment (scale 1-5).
    • 1 = Excellent
    • 2 = Very good
    • 3 = Good
    • 4 = Fair
    • 5 = Poor
16. MentHlth- Number of days with poor mental health in the last 30 days.
    • Scale 1-30 days
17. PhysHlth- Number of days with poor physical health in the last 30 days.
    • Scale 1-30 days
18. DiffWalk- Difficulty walking or climbing stairs.
    • 0 = No difficulty
    • 1 = Yes, difficulty present
19. Sex
    • 0 = Female
    • 1 = Male
20. Age- Age category (13 levels).
    • 1 = 18-24 years
    • 2 = 25-29 years
    • 3 = 30-34 years
    • 4 = 35-39 years
    • 5 = 40-44 years
    • 6 = 45-49 years
    • 7 = 50-54 years
    • 8 = 55-59 years
    • 9 = 60-64 years
    • 10 = 65-69 years
    • 11 = 70-74 years
    • 12 = 75-79 years
    • 13 = 80 years or older
21. Education- Education level (scale 1-6).
    • 1 = Never attended school / kindergarten only
    • 2 = Elementary school (grades 1-8)
    • 3 = Incomplete high school (grades 9-11)
    • 4 = High school graduate / GED
    • 5 = 1-3 years of college/technical school
    • 6 = College graduate
22. Income
    - 1 = Less than $10,000
    - 2 = $10,000 to less than $15,000
    - 3 = $15,000 to less than $20,000
    - 4 = $20,000 to less than $25,000
    - 5 = $25,000 to less than $35,000
    - 6 = $35,000 to less than $50,000
    - 7 = $50,000 to less than $75,000
    - 8 = $75,000 or more
