# Supplementary figures and images for: AI-driven analysis of diabetes risk determinants in U.S. adults: Exploring disease prevalence and health factors
Source: PLoS One. 2025 Sep 3;20(9):e0328655. doi: 10.1371/journal.pone.0328655 (PMC12407459; doi:10.1371/journal.pone.0328655)

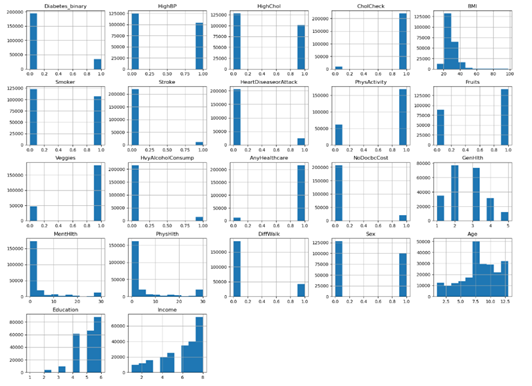

Supplement: S1 Fig — (PNG) [file pone.0328655.s002.png]

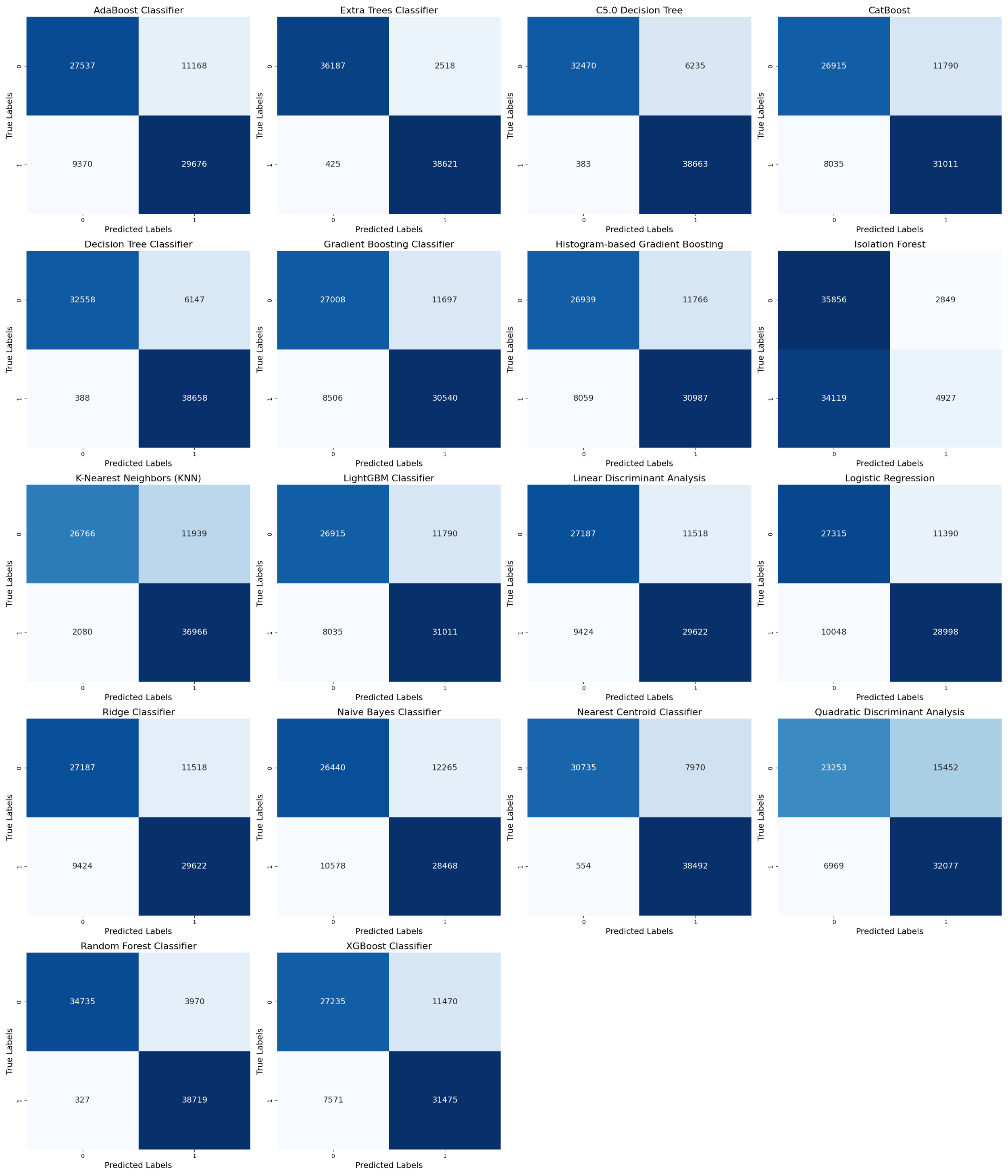

Supplement: S3 Fig — (PNG) [file pone.0328655.s004.png]
