# Supplementary material for: AI-driven analysis of diabetes risk determinants in U.S. adults: Exploring disease prevalence and health factors
Source: PLoS One. 2025 Sep 3;20(9):e0328655. doi: 10.1371/journal.pone.0328655 (PMC12407459; doi:10.1371/journal.pone.0328655)
Supplement: S1 Table — (DOCX) [file pone.0328655.s005.docx]

**S1 Table. Comparison of effectiveness of different techniques for unbalanced dataset.**

| Model | Technique | Accuracy (%) | Precision (Diabetes Class) | Recall (Diabetes Class) | AUC / R² / MSE |
| --- | --- | --- | --- | --- | --- |
| Extra Trees Classifier | ROS | 0.96 | 0.94 | 0.99 | 0.96 |
|  | SMOTE | 0.95 | 0.96 | 0.96 | 0.95 |
|  | Adasyn | 0.94 | 0.96 | 0.93 | 0.94 |
| C5.0 Decision Tree | ROS | 0.92 | 0.99 | 0.99 | 0.92 |
|  | SMOTE | 0.93 | 0.94 | 0.94 | 0.93 |
|  | Adasyn | 0.90 | 0.94 | 0.9 | 0.90 |
| Decision Tree Classifier | ROS | 0.92 | 0.99 | 0.99 | 0.92 |
|  | SMOTE | 0.93 | 0.94 | 0.94 | 0.93 |
|  | Adasyn | 0.90 | 0.94 | 0.89 | 0.90 |
| Random Forest Classifier | ROS | 0.90 | 0.87 | 0.96 | 0.90 |
|  | SMOTE | 0.95 | 0.96 | 0.95 | 0.95 |
|  | Adasyn | 0.94 | 0.96 | 0.93 | 0.94 |
